# Precision Nutrition for Targeting Lipid Metabolism in Colorectal Cancer

**DOI:** 10.3390/nu9101076

**Published:** 2017-09-28

**Authors:** Cristina Aguirre-Portolés, Lara P. Fernández, Ana Ramírez de Molina

**Affiliations:** Molecular Oncology and Nutritional Genomics of Cancer Group, IMDEA Food Institute, CEI UAM + CSIC, Carretera de Cantoblanco 8, E-28049 Madrid, Spain; cristina.aguirre@imdea.org (C.A.-P.); lara.fernandez@imdea.org (L.P.F.)

**Keywords:** precision nutrition, lipid metabolism, colorectal cancer, diet, genomics, transcriptomics, SNPs, obesity, microbiota

## Abstract

Cancer is a multistage and multifactorial condition with genetic and environmental factors modulating tumorogenesis and disease progression. Nevertheless, cancer is preventable, as one third of cancer deaths could be avoided by modifying key risk factors. Nutrients can directly affect fundamental cellular processes and are considered among the most important risk factors in colorectal cancer (CRC). Red and processed meat, poultry consumption, fiber, and folate are the best-known diet components that interact with colorectal cancer susceptibility. In addition, the direct association of an unhealthy diet with obesity and dysbiosis opens new routes in the understanding of how daily diet nutrients could influence cancer prognosis. In the “omics” era, traditional nutrition has been naturally evolved to precision nutrition where technical developments have contributed to a more accurate discipline. In this sense, genomic and transcriptomic studies have been extensively used in precision nutrition approaches. However, the relation between CRC carcinogenesis and nutrition factors is more complex than originally expected. Together with classical diet-nutrition-related genes, nowadays, lipid-metabolism-related genes have acquired relevant interest in precision nutrition studies. Lipids regulate very diverse cellular processes from ATP synthesis and the activation of essential cell-signaling pathways to membrane organization and plasticity. Therefore, a wide range of tumorogenic steps can be influenced by lipid metabolism, both in primary tumours and distal metastasis. The extent to which genetic variants, together with the intake of specific dietary components, affect the risk of CRC is currently under investigation, and new therapeutic or preventive applications must be explored in CRC models. In this review, we will go in depth into the study of co-occurring events, which orchestrate CRC tumorogenesis and are essential for the evolution of precision nutrition paradigms. Likewise, we will discuss the application of precision nutrition approaches to target lipid metabolism in CRC.

## 1. Introduction

Cancer is the second leading cause of mortality and is responsible for one sixth of deaths worldwide. During 2015, there were 17.5 million cancer cases and 8.8 million patient’s deaths [1]. Particularly, colorectal cancer (CRC) ranks as the third leading cause of cancer-related deaths (data from The World Health Organization; WHO). In the course of this multifactorial condition, a cascade of alterations takes place, modifying the expression of both tumor suppressor genes and oncogenes. Together with this, when compared to quiescent cells, proliferating cells present a distinct metabolism characterized by high rates of glycolysis, lactate production, and the biosynthesis of lipids and other macromolecules. During the last decade, many laboratories focused their interest on understanding this metabolic switch that occurs during tumorogenesis [2,3]. In fact, several studies have demonstrated the importance of lipid metabolism regulation in the promotion of migration [4], invasion [5,6], and angiogenesis [7,8], three basic steps during metastasis [9]. Regarding CRC, key enzymes involved in lipid-metabolic pathways have been found differentially expressed in normal and tumoral tissues. Some of them were associated with cancer survival and were individually proposed as prognosis markers [6,10,11]. Furthermore, one of the transcriptomic consensus molecular subtypes (CMS) of CRC described by Guinney and colleagues [12], the “metabolic subtype 3” (CMS3), exhibits a clear enrichment for multiple metabolism signatures along with KRAS (Kirsten Rat Sarcoma Viral Oncogene Homolog)-activating mutations that have been described as inducing metabolic reprogramming [12]. 

**Figure 1:**
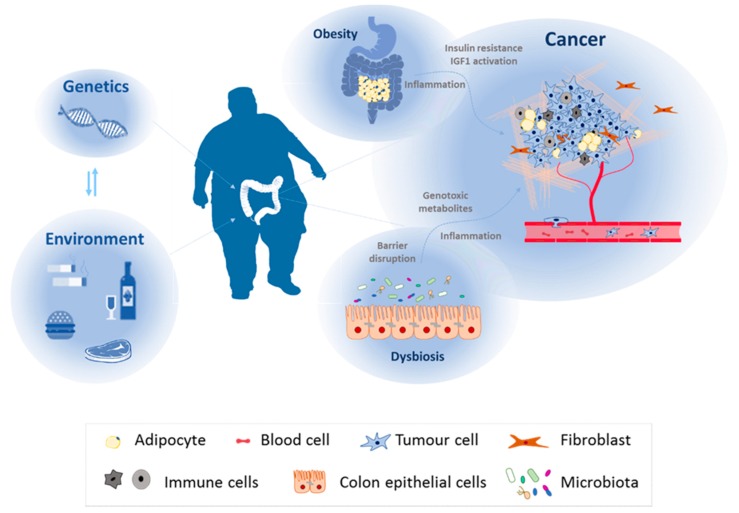
Colorectal cancer malignancy relays on genetic factors, environmental factors, and their interaction between each other. Together with patient genome, environmental factors associated with lifestyle (alcohol consumption, smoking, unhealthy diet, or reduced physical activity) influence colorectal cancer initiation and malignancy. They can alter specific target tissues or affect human physiology, giving rise to pathologies that can promote tumour progression such as obesity or dysbiosis.

The majority of the primary tumours initially respond to chemotherapy and regress, but frequently and due to minimal residual diseases, they relapse and are no longer sensitive to therapy [13]. The genetic alterations that directly affect the genome of tumoral cells before diagnosis and during treatment are the most studied factors implicated in resistance acquisition. However, not only gene expression but the interaction between genetic factors and environment plays a crucial role in the causality of cancer progression [14,15] (Figure 1). In this direction, epigenetic changes that could be originated by environmental factors can provide tumour heterogeneity and an ineffective response to chemotherapy [16]. 

Tumours cannot be considered as simple bulks of cells with an altered cell cycle control. Neoplastic cells interact with each other and, more importantly, with the healthy tissue that surrounds them, the tumour microenvironment (TME) [17]. Thus, during the neoplastic progression, a co-evolution takes place between malignant cells, the extracellular matrix (ECM), cancer associated fibroblasts (CAF), the immune system, and the vascular endothelial cells. Tumor heterogeneity not only relies on intratumoral variability derived from Darwinian evolution [18], but also on the different types of immune cells that infiltrate the primary tumor, the plasticity of the CAFs and the tissue of origin responsible for the neo-vascularization [19]. Distant metastases are responsible for 90% of cancer deaths, making it essential to consider its phenotypic variability in cancer malignancy (reviewed by Marusyk et al. [18]). Based on this intra-tumoral, TME and distant metastasis heterogeneity, basic research together with translation medicine and oncologists in the clinic, are gathering strength to identify patient subpopulations and designing new targeted therapies for personalized treatment. 

Current evidence demonstrates that one third of cancer deaths could be prevented by modifying key risk factors such as smoking, assessment of infection-related risk factors, or alcohol consumption. Moreover, physical activity, nutrition, and diet are also considered among the most important environmental risk factors for cancer development due to their association with obesity (Figure 1). Nutrient components can modulate cancer progression or even the risk of developing this disease by regulating, directly or indirectly, gene expression. 

In the early seventies, red meat and fat consumption were already proposed to increase the incidence and mortality of colon cancer [20,21]. Furthermore, in the last decade, several studies based on meta-analyses demonstrated a link between obesity, risk of cancer, and disease prognosis [22].

The recent development of powerful “omics” technologies has opened new avenues towards nutritional sciences. The genomics, transcriptomics, proteomics, metabolomics, and lipidomics approaches lead to a new vision of the delivery of nutritional advice: the precision nutrition. Current views on precision nutrition consider this discipline at three stages: (1) conventional nutrition based on general guidelines for population groups; (2) individualized nutrition based on phenotypic information; and (3) genotype-directed nutrition focused on gene variation and its consequences [23]. 

Single nucleotide polymorphisms (SNPs) are the most common forms of sequence variation in the human genome [24]. The analysis of SNPs is a well-known tool for precision nutrition, and the recent development of next generation sequencing (NGS) techniques is highly improving genetic variation studies [23,25]. Likewise, transcriptomic studies and RNA sequencing analyses will also refine precision nutrition. So far, both genomic and transcriptomic studies have been extensively used in precision nutrition approaches. Finally, the future technical development of proteomics, metabolomics, and lipidomics will complete the full nutritional landscape [23]. 

In this review, we will discuss co-occurring events that take place in the course of tumorogenesis and that were essential for the evolution of precision nutrition paradigms. The importance of new “omics” technologies such as genomics and transcriptomics and their application to target lipid metabolism will also be detailed.

## 2. Lipid Metabolism, Diet and Colorectal Cancer

Metabolic alterations encountered in tumors are well described and considered as a hallmark of cancer [26]. Taking into account the importance of lipids at different levels in cellular physiology, alterations in fatty acids (FA) synthesis and lipid metabolism can interfere with very diverse cellular processes that go from plasmatic and organelles membrane organization and plasticity [27,28], substrate supply for ATP synthesis [29], to cell signaling activation [30]. 

It is important to mention that these alterations do not only affect the primary tumour in a cell autonomous manner, the exogenous lipids synthetized by tumour microenvironment could also influence malignancy [31,32,33,34]. For example, pro-inflammatory eicosanoids can directly promote cell proliferation, apoptosis, migration and invasion. More importantly, they are also associated with angiogenesis promotion [35]. 

Regarding the primary tumour, alterations in lipid metabolism-related genes are able to promote migration and invasion. TGFβ was shown to promote epithelial-to-mesenchymal transition (EMT) together with a lipogenesis suppression, favoring energy production [4]. While sphingosine 1-phosphate plays an inhibitory role, lysophosphatidic acid (LPA) is able to regulate Membrane-type matrix metalloproteinase 1 (MT1-MMP) and promote invasion [5]. In addition to that, a recent publication uncovered ACSL1 (an isozyme of Acyl-CoA synthetase) and SCD (Stearoyl-CoA-desaturase 1) as part of a metabolic network that increased energetic efficiency in CRC-derived cells together with the promotion of migratory and invasive capacity [6]. Moreover, both Sphingosine 1-phosphate signaling pathways [36] and CPT1A (Carnitine Palmitoyltransferase 1A), a rate-controlling enzyme in fatty acid β-oxidation [8], were associated with lymphangiogenesis. 

Several categories of lipids have been studied regarding its association with CRC. Fatty acids are the building blocks for the formation of more complex lipids and they have also been associated with colorectal tumorogenesis. Importantly, not all FAs behave in the same direction. First, plasma concentration of saturated FA (SFA) as well as essential FAs were found to be significantly decreased in CRC patients when compared to healthy controls [37]. Moreover, while ω3 is associated with a protective role in CRC [38,39,40], other types of polyunsaturated FA (PUFA), ω6 present opposite effects [41]. Regarding dietary FAs, while intake of unsaturated FA (UFA) may be beneficial for health [42], SFAs were associated with tumorogenesis [41,43]. The steroids are essential components of membrane lipids and can act as signaling molecules. Very low-density lipoprotein cholesterol (VLDL) was shown to be positively correlated with adenoma frequency in colon. Importantly, triglycerides (TG) and LDL were associated CRC prognosis, as its significantly increased levels were found in patients with distant metastasis. Cholesterol is present in high-fat diets and, together with red meat and total fat, its consumption is strongly associated with colorectal tumorogenesis [44]_._ Other essential structural components of the cellular membrane are the sphingolipids. Among them, ceramide is known as a chemopreventive agent; in fact, used in combination with tamoxifen, it is able to arrest cell cycle progression and promote apoptosis [45]. Glycerophospholipids are the major lipid components of the cellular membrane. The expression of cyclic phosphatidic acid (cPA) was found to impair metastasis and invasion of cancer cells [46]. However, phosphatidylcholine (PC) was found significantly increased in CRC-derived cells [47]. The knowledge of how glycerolipids, key molecules for the synthesis of membrane lipids, and TG could influence CRC remains shallow, but a protective role was described for 1-*O*-octadecyl 2-*O*-methyl-sn-glycerophosphocholine [48]. 

In addition to genetic alterations in genes that regulate lipid metabolism, affecting directly colorectal tumorogenesis, an unhealthy diet would be able to modify the physiology of the patients, giving rise to comorbidities that promote tumour growth and invasion of distant tissues [49].

### 2.1. Diet, Obesity, and CRC

Obesity can appear as a result of an unbalanced diet where the caloric intake is higher than the energy expenditure. This pathology is defined by an excessive adipose tissue accumulation that associates with a risk to the health of an individual [22]. Importantly, the worldwide prevalence of this pathology has doubled between 1980 and 2014. Nowadays, 13% of the overall adult population worldwide suffer from obesity. Importantly, in 2014, 41 million children under the age of 5 years were overweight or obese (WHO) and, by 2030, the number of overweight and obese adults is projected to reach 2.16 billion. Therefore, an unquestionable cause of concern is the constant increase in childhood obesity. The dietary patterns of children from low- and middle-income countries together with the low levels of physical activity gave rise to a sharp increase in obesity (WHO) [17]. This pathology has been implicated in the development of cardiovascular diseases and type-2 diabetes [50] as well as in the initiation and dissemination of several types of cancer [51]. In fact, overall risk of death from cancer is 1.5–1.6-fold higher in men and women with a BMI > 40 kg/m [52]. The excess of visceral fat provokes alterations in the cellular composition of the adipose tissue and promotes the increased malignancy of tumors that develop in a microenvironment rich in adipocytes like breast, ovary, or colon tumors [53]. The main types of cancer whose increased risk has been associated with obesity are: prostate [54], postmenstrual endometrial [55] and breast [51], ovary [56], bladder [57], liver [58], colon [22], pancreas [51], esophageal [59], gallbladder [60], kidney [61], and thyroid cancer [62]. 

During the last decade, in parallel with a decreased physical activity, the caloric intake has constantly increased. The main environmental factors that interact with genetic variants and contribute to obesity are sugar-sweetened beverages, fried food consumption, and sedentary lifestyle [63]. As a direct consequence of this energetic imbalance, a metabolic shift takes place in the body, giving rise to hypertrophy and hyperplasia of the adipose tissue. 

In the course of obesity, the excess of adipocytes accumulates in locations not classically associated with adipose tissue. This increase in systemic ectopic fat shows positive correlation with several types of cancer, CRC among them [64]. Currently, there are two wide-spread hypotheses describing the underlying molecular mechanisms that link obesity and colorectal cancer: (1) insulin resistance and the activation of insulin growth factor-1 (IGF1). A large volume of epidemiological studies as well as meta-analysis, driven independently by several groups, demonstrated that the total levels of this growth factor correlates to several types of cancer [62,65,66,67]; (2) systemic inflammation due to hypertrophy of adipose tissue [68] (Figure 1). 

Both overweight and obesity are largely preventable, but social education for health promotion, the individual responsibility, as well as the food industry, together with basic research, need to team up to face this urgent global health challenge. 

### 2.2. Diet, Dysbiosis, and Colorectal Cancer

The term human microbiota refers to the assemblage of microorganisms (bacteria, archaea, or lower eukaryotes) present in a defined environment, such as the gastrointestinal tract. It consists of the 10–100 trillion symbiotic microbial cells harbored by each person, primarily bacteria in the gut. A symbiotic association with microbiota exists in healthy individuals, offering protection from invading pathogens and preventing tumorogenesis (eubiosis). The pathological condition developed when the gut microbiota homeostasis is disturbed, due to an imbalance in the flora, changes in functional composition and metabolic activities or changes if their local distribution is defined as dysbiosis [69]. This pathological scenario is characterized by a decrease in microbial diversity and an increase in pro-inflammatory species. This imbalanced microbiota is unable to protect from pathogenic organisms that could successfully be established and trigger inflammation, as well as producing genotoxins and carcinogenic microbial metabolites (Figure 1). 

Moreover, during obesity and along with its comorbidities, the composition of gut microbiota and the features of the intestinal epithelium are altered, affecting its barrier function. Among other diseases, microbial dysbiosis was associated with colorectal carcinogenesis and gastric and esophageal cancers [70,71]. Several diet components are known to influence microbiota and protect or cause detrimental metabolites that negatively affect the digestive tract. Therefore, CRC can be influenced not just by specific pathogens in the patient but also by the metabolic output of the entire microbiota [72]. Both high fat diets as well as low fiber intake can lead to dysbiosis [72]. Low-carbohydrate intake or an extreme change from vegetable-based to animal-based diet would drastically affect microbiota composition. In fact, variations in regular diet generate specific profiles of microbiota: the pathogens present in the microbiota of an individual with high consumption of fiber would be different if we compared them with the composition of patients with high protein and fat intake [72]. These profiles, indeed, determine CRC incidence in different populations due to changes in dietary patterns, as they occur when rural native Africans are compared with African Americans [73]. 

The etiology of CRC is partially dependent on microflora and diet, so nowadays there is increasing interest in the use of probiotics to modulate gut microbiota [74]. The Food and Agriculture Organization of the United Nations (FAO) defines “probiotics” as the only bacterial group classified as functional food which is intended to be consumed as part of a normal diet and that delivers biologically active components that have the potential of disease risk reduction. The exact mechanisms underlying this positive association between probiotics and health are not fully understood, but there are consistent epidemiological and experimental data supporting its positive association with CRC prevention and treatment [75]. There are several proposed effects that would explain the anticancer effects of probiotics: (1) lowering of intestinal pH; (2) inactivation of carcinogens; (3) modulation of immune cells populations; (4) modulation of the physical barrier by altering the intestinal microflora; (5) modulation of apoptosis and cell proliferation [74]. Epidemiological studies have shown that the composition of CRC patients microbiota is different to the one present in healthy population [76]. Taking into account the effectiveness of probiotics in modulating microbiome composition, the design of microbiota-targeting therapies is now considered as a feasible strategy in the clinic, both as a preventive and a therapeutic approach [77]. 

## 3. Nutrition and Colorectal Cancer

Although the 5-year survival after CRC diagnosis when metastasis is already present has improved in the last decade, this decrease is still lower than 3% [78]. The sequential administration of three different chemotherapeutic drugs, along with vascular endothelial growth factor (VEGF) and epithelial growth factor receptor (EGFR) inhibitors, allowed the median survival of the patients to reach 30 months [79]. 

In most cases, early detection allows tumors to be successfully removed by surgery and increases treatment efficiency. Nowadays, great part of survival improvements rely on CRC screening programs that allow early diagnosis [12]. Thus, the main challenge in the clinic nowadays is the understanding and characterization of the CRC inter and intra-tumour heterogeneity originated by genomic, epigenomic, transcriptomic, and immune variability to stratify patients and shape the future clinical development of personalized treatments [80]. New technologies allowed the identification of new biomarkers and their co-evolution with drug discovery and targeted therapies design. Importantly, cancer susceptibility does not just depend on the genetic background of patients; environmental factors as well as lifestyle are determinants in the etiology of CRC [81]. Despite the improvements in early diagnosis and targeted therapies design for CRC, the rates of its incidence have been increasing for people younger than 40 years, pointing out the pressing needs for identifying the underlying environmental factors and providing preventive strategies for high-risk individuals [82]. In addition to alcohol consumption, smoking [83] or the presence of dysbiosis [84], diets rich in red, processed, and grilled meats were strongly associated with colorectal cancer [85,86]. Several studies published during the last two years supported the assessment published by the International Agency for Research on Cancer (IARC) in October 2015 (data summarized by Bouvard et al. [87]). The results gathered by IARC reached the final conclusion that the consumption of 50 g of red meat per day increased by 18% the risk of suffering CRC. The meta-analysis carried out by Carr and collaborators found that different red meat subtypes influence differently the diverse CRC subtypes [88]. Moreover, they demonstrated that, although poultry or pork intake was not associated with higher risk of CRC, beef and lamb consumption presented positive moderate association. Importantly, in a second publication by the same group, they analysed patients 5 years after diagnosis and they observed no increased mortality in those with higher red and processed meat intake [89]. When pre-diagnosed consumption of red, processed meat, and poultry was assessed, no relation to CRC survival was found for red meat, whereas positive association was demonstrated for processed meat and CRC mortality in females. An increased all-cause mortality was associated with poultry consumption. Finally, no changes in CRC mortality were found for dietary fiber [90].

Regarding the molecular mechanisms underlying this detrimental effect of red and processed meat, multiple components were implicated. First, an increase in N-nitroso components production is induced in the digestive tract by red and processed meat consumption. Moreover, two genotoxic compounds that cause DNA damage as heterocyclic aromatic amines and poly-cyclic aromatic hydrocarbons are present in high-temperature cooked meat and smoked or grilled meat, respectively. In 2013, results from Kentucky Colon Cancer Study established statistical correlations among total dietary intake of 2-amino-3,8-dimethylimidazo[4,5-f]quinoxaline, 2-amino-3,4,8-trimethylimidazo[4,5-f]quinoxaline, meat-derived mutagenic (a marker for meat mutagens combined) and colon cancer risk. Their analysis support estimated heterocyclic amines and polycyclic aromatic hydrocarbons exposure as being a possible mechanism to increase colon cancer risk in the context of red meat intake [91]. Besides, higher oxidative stress, as well as induction of *APC* gene mutations and its promoter methylation, were suggested to be triggered by increased red and processed meat intake, and therefore with CRC risk [86,87].

Fiber is also among the well-studied dietary factors associated with colorectal tumorogenesis. In this case, most of the epidemiological data defend the protective role of dietary fiber in CRC, but the data are still no conclusive and further research needs to be performed [83,90,92]. 

Considering specific nutrient components, folate has been deeply studied as a modulator of colorectal cancer prevention [93,94]. Plasma alterations of this water-soluble vitamin B_9_ are associated with the hypermethylation of several tumour suppressor genes and with DNA hypomethylation [95]. The most consistent piece of data demonstrating that folate can be considered an independent risk factor for CRC was published in 2011 [96]. The authors performed the largest prospective cohort study in this regard and showed that those individuals with the highest folate intake presented a 30% reduction in the risk of developing CRC. 

In addition, according to several epidemiologic studies, milk, calcium and dietary vitamin D are considered as protective factors against CRC development and are positively associated with survival [97,98].

Apart from specific nutrients, the direct association of diet with an excessive accumulation of adipose tissue and the subsequent development of obesity plays a role in tumor prognosis. During cancer progression, a bidirectional crosstalk is established between malignant cells and adipocytes [78]. Because of malignant cell proximity, the cancer associated adipocytes (CAA) suffer delipidation and acquire fibroblast-like features that will influence malignancy. The lipids secreted by adipocytes are transferred to cancer cells that can use them for energy production through β-oxidation. Moreover, the rapid expansion and hypertrophy of adipose tissue provokes oxygen deficiency, and compensatory mechanisms to promote angiogenesis are triggered, favoring tumor spreading [99]. 

### Precision Nutrition in Colorectal Cancer 

The relation between CRC carcinogenesis and nutrition factors is probably more complex than originally conceptualized. However, it is widely accepted in the field of precision nutrition that several genetics variants in diet-nutrition-related genes are clearly associated with CRC prevention (Table 1) [49]. The most representative example is the association between variants in genes related to folate synthesis and CRC risk. Folate is involved in the synthesis of nucleic acids and DNA methylation [100]. It has been described that genetic polymorphisms in methylenetetrahydrofolate reductase (MTHFR) enzyme are modulating their own activity. In addition, SNPs in *MTHFR* and levels of folate intake combine to regulate CRC risk [100,101]. Particularly, minor homozygous allele TT of Cys677Thr polymorphism in *MTHFR* gene reduces in vitro MTHFR enzymatic activity to 30%. The TT genotype is associated with CRC risk in the context of low folate intake, whereas it is protective for CRC when high intake of folate occurs [100,101,102].

Many other examples of polymorphisms in diet-nutrition and/or metabolism-related genes, that modulate CRC risk, have been described in the literature. Genetic variations in enzymes like glutathione S-transferases (GSTs) have been related to CRC risk. These proteins are involved in phase II detoxification process of drugs and endogenous compounds. *GSTM1* and *GSTT1* null genotypes increased risk of CRC in Caucasian populations [103,104,105]. Polymorphisms in vitamin D receptor (*VDR*) gene, in combination with dietary fat or calcium, seem to also modulate CRC risk, but controversial results have been found [106,107,108,109]. The common Thr1482Ile polymorphism in the transient receptor potential melastatin 7 (*TRPM7*), a ubiquitously expressed constitutive ion channel with higher affinity for Mg2+ than for Ca2+, was associated with an elevated risk of both adenomatous and hyperplastic polyps. Moreover, this polymorphism significantly interacted with the Ca:Mg intake in relation to both types of polyps [110]. SNPs in genes belonging to the base excision repair pathway (BER) have been associated with CRC risk. An association has been reported between Glu51His in *APEX1* (ascorbate peroxidase) with CRC risk and a modifier role for the Val762Ala SNP in *PARP* gene (poly ADP ribose polymerase) on the effect of diets higher in high-temperature cooked red meat [111]. Genes belonging to angiogenesis pathway have been interrogated for gene-environment interactions: SNPs and smoking, dietary protein, and alcohol exposures, as well as associations of these interactions with CRC risk and survival. Variants on *FLT1* (vascular endothelial growth factor receptor 1) interacted with smoking, animal protein intake, and CRC risk. Besides, *KDR* (vascular endothelial growth factor receptor 2) variants interacted with alcohol and CRC risk [112]. However, there is high inter-group variability in the results. Replication studies with accurate designs are needed in order to clarify the use of many of these markers before applying these results to clinical practices [113,114]. In this context, consortium studies arise in order to solve reproducibility and low sample size problems [24]. Huge collaborations among scientific groups, such as the Personal Genome Project, the International HapMap consortium, or the Human Variome Project have been established to obtain information on genetic variation with the goal of linking genetic variation to human disease risk and promoting the development of personalized medicine [25].

Together with genomic approaches, transcriptomic studies also have been used for precision nutrition in order to study responses to nutrients and/or bioactive products that can influence gene expression. In this transcriptomic scenario, a representative example of a metabolic gene whose expression is both dysregulated in CRC and modulated by bioactive compounds is *GCNT3* (Glucosaminyl (*N*-Acetyl) transferase 3, mucin type)*.* It codifies for a glycosyltransferase enzyme implicated in glycosylation processes. *GCNT3* catalyzes the formation of core 2 *O*-glycan, core 4 *O*-glycan, and I branches in mucin-type glycoproteins biosynthesis [115]. *GCNT3* expression is altered in cancer [116,117,118,119] and its upregulation has been clearly associated with favorable CRC prognosis [116,117].

It has been established that rosemary extracts regulate *GCNT3* expression in CRC. Rosemary (*Rosmarinus officinalis* L.) is an evergreen shrub from Mediterranean region [120]. The rosemary leaves have been employed as seasoning as well as in traditional medicine for treating several disorders such as renal colic and respiratory diseases. In recent years, scientific investigations have been performed in order to elucidate the potential utility of rosemary extracts and/or their constituents with antioxidant activity in several diseases, including cancer [120,121]. The major components of rosemary extracts are carnosic acid, carnosol, ursolic acid, and rosmarinic acid. Some of them exhibit intrinsic antitumor properties; nevertheless, the efficacy of the complete extract is usually higher, due to a synergistic effect as well as the presence of additional antitumor components whose effect has not been demonstrated yet. Moreover, rosemary extract has also been used in combination with several antitumor agents and chemotherapeutic drugs [121].

Interestingly, it has been shown that rosemary was able to increase the expression of *GCNT3* gene. *GCNT3* upregulation is associated with better prognosis in CRC [116,117] and this upregulation correlated with the antiproliferative effect of different rosemary extracts in tumor cells [122]. Moreover, rosemary also regulates miR-15b expression, which was reported to target *GCNT3* by *in silico* analysis [122]. miR-15b has been found upregulated in CRC patients and, consequently, it has been considered as potential biomarker [123]. miR-15b expression was downregulated by rosemary in CRC cells. The rosemary component responsible for this modulation is carnosic acid. Since this regulation was also detected in plasma, miR-15b could be considered as a potential non-invasive biomarker to monitor in vivo responses [122].

## 4. Precision Nutrition and Lipid Metabolism in Colorectal Cancer

In 2015, consensus studies on transcriptomic data were successfully applied in order to solve the intrinsic heterogeneity and molecular complexity of CRC. Several international research groups shared large-scale data and they proposed a new CRC classification based on an unbiased approach to facilitate clinical translation [12]. They established four transcriptomic consensus molecular subtypes (CMS) of CRC: CMS1 or microsatellite instability (MSI) immune subtype, CMS2 or canonical, CMS3 or metabolic, and CMS4 or mesenchymal subtype. 

Interestingly, metabolic CRC subtype tumours (CMS3) have been characterized as those which harbor *KRAS* mutations, a mixed MSI status, low somatic copy number alterations (SCNA), and low CpG island methylator phenotype (CIMP). Furthermore, CMS3 tumours exhibit a prominent metabolic activation with a clear enrichment for multiple metabolism signatures, in connection with the presence of *KRAS*-activating mutations that have been described as inducing metabolic reprogramming [12]. Although *KRAS* mutants are more prevalent among CMS3 tumours, they are present in every molecular subtype. *KRAS* mutations were more likely to be present in patients without a family history of colon cancer and never smokers [124,125]. In a meta-analysis performed in 2009, no association was observed between smoking and *KRAS* mutations in colorectal adenocarcinomas [126]. A recent study links alcohol intake with an increased risk of *KRAS*+ and *BRAF*-/*KRAS*- [127]. Furthermore, a positive association was reported between heme iron intake from red meat and the risk of CRC with activating G > A mutations in *KRAS* [128].

**Figure 2:**
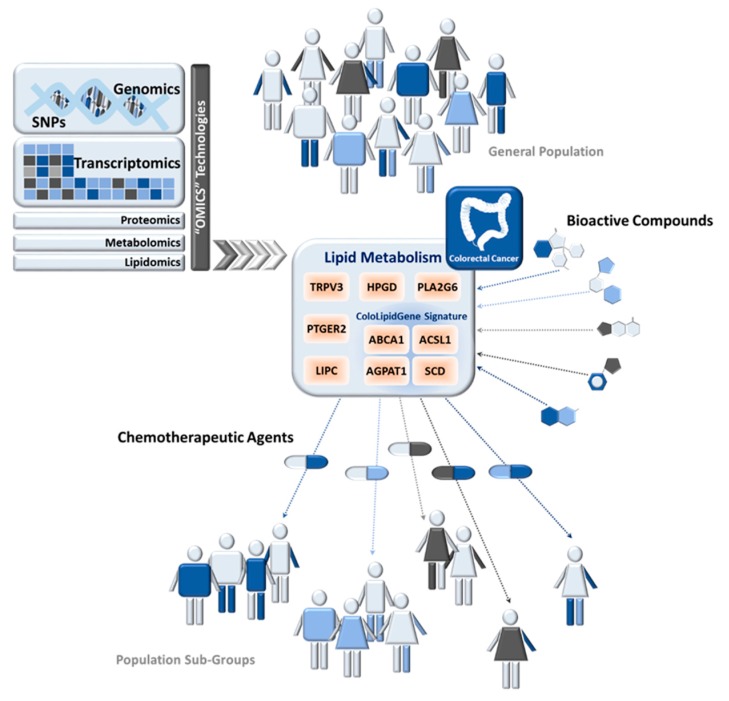
Modulation of Lipid Metabolism in Colorectal Cancer by Precision Nutrition Approaches. Genomics, transcriptomics, and other “omics” technologies have significantly contributed to the development of precision nutrition, which aims to identify patient subpopulations and design new targeted strategies for personalized treatment. Alterations in lipid metabolism have been implicated in cancer-metabolic progression. Examples of lipid-metabolic genes that have been interrogated for precision nutrition uses in colorectal cancer (CRC) are detailed. Bioactive compound could modulate lipid-metabolism-related gene expression. Their use together with classical chemotherapeutic agents, whose effect could be potentiated, is one of the current lines of research in CRC treatment. *HPGD*: hydroxyprostaglandin dehydrogenase 15-(NAD), *PLA2G6*: phospholipase A2 group VI, *TRPV3*: transient receptor potential vanilloid 3, *PTGER2*: prostaglandin E receptor 2, *LIPC*: hepatic triglyceride lipase, *ACSL1*: Acyl-CoA synthetase 1, *ABCA1*: ATP-Binding Cassette Subfamily-A Member 1, *AGPAT1*: 1-Acylglycerol-3-Phosphate *O*-Acyltransferase 1, *SCD*: Stearoyl-CoA-desaturase 1, SNPs: Single Nucleotide Polymorphisms.

In this cancer-metabolic scenario, the examples of metabolism-related pathways that could be implicated in precision nutrition are currently increasing. Alterations in lipid metabolism also contribute to cancer-metabolic progression (Figure 2). Highly proliferative cancer cells display strong lipid and cholesterol avidity, which they satisfy by increasing the uptake of dietary or exogenous lipids and lipoproteins or activating lipogenesis or cholesterol synthesis [129]. 

Metabolic genes belonging to fatty acids synthesis pathway have been interrogated in CRC for precision nutrition uses (summarized in Table 2). In 2010, 43 fatty acid metabolism-related genes and 392 SNPs were analyzed in 1225 CRC cases and 2032 controls from the European Prospective Investigation into Cancer and Nutrition study (EPIC cohort) [130]. Authors found evidence for an association of hydroxyprostaglandin dehydrogenase 15-(NAD) (*HPGD*), phospholipase A2 group VI (*PLA2G6*), and transient receptor potential vanilloid 3 (*TRPV3*) with increased risk for CRC, while prostaglandin E receptor 2 (*PTGER2*) was associated with lower CRC risk. This work highlighted the role of prostanoid signaling in colon carcinogenesis and gave weight to the relevance of genetic variation in fatty acid metabolism-related genes and CRC risk [130]. After that, a different study analyzed a new set of 8 fatty acid biosynthesis-related genes (30 SNPs) in 1780 CRC cases and 1864 controls from the Molecular Epidemiology Cancer study [131]. They found an association of rs9652472 polymorphism of *LIPC* (hepatic triglyceride lipase) with increased risk of CRC. They also replicated previous associations of *LIPC* SNPs with higher serum HDL levels [131]. 

Recently, genetic analysis of 57 SNPs located in 7 lipid-metabolism-related genes was performed in CRC patients in order to identify whether any genetic alteration might be related to overexpression of these enzymes and therefore constitute a biomarker of lipid metabolism-related alterations [11]. In a multivariate model, adjusting for clinical risk factors and multiple comparisons, the SNP rs8086 in *ACSL1* was associated with CRC disease-free survival (DFS), indicating that patients carrying the *ACSL1* rs8086 T/T genotype had significantly decreased DFS compared with patients carrying the C/T or C/C genotype, with 3-fold higher risk of relapse (Table 2). T/T genotype for rs8086 is associated with worse clinical outcome and simultaneously correlates with high ACSL1 mRNA levels, which, in turn, had already been associated with worse clinical outcome in these CRC patients [10,11]. Previous to this study, a lipid-metabolic signature (ColoLipidGene) was associated with CRC prognosis in stage II patients [10]. ColoLipidGene signature encompasses the transcriptional activation of four metabolic-related genes: *ACSL1*, *ABCA1* (ATP-Binding Cassette Subfamily-A Member 1), *AGPAT1* (1-Acylglycerol-3-Phosphate *O*-Acyltransferase 1), and *SCD*. Results from three different groups of patients, together with data from publicly available repository GEO (Gene Expression Omnibus Database), point out the activation of *ABCA1*, *ACSL1*, *AGPAT1,* and *SCD* as one of the main relevant metabolic factors in CRC malignant progression [10].

The extent to which genetic variants, together with intake of specific dietary components, affect risk of CRC in different populations is currently under investigation. Several natural compounds may modulate lipid metabolism [132] and, consequently, they could have a key role in the prevention and treatment of cancer [133,134,135,136]. Indeed, many anticancer agents employed clinically are natural compounds or their derivatives. Various in vitro studies pointed out an external regulation of lipid-metabolism-related genes whose expression could be modulated by bioactive products. Moreover, the use of bioactive compound together with classical chemotherapeutic agents, whose effect could be potentiated, constitutes an important line of development in CRC treatment with increasing number of clinical studies trying to address this point (Figure 2).

In the context of ColoLipiGene signature, few studies are focused on the transcriptional regulation of genes belonging to this signature by bioactive compounds that must be further explored for therapeutic or preventive application. For example, it is known that in THP-1 cells, eicosapentaenoic acid (EPA)-rich oil altered the expression of fatty acids metabolism genes including *SCD* and FA desaturase-1 and -2 (*FASDS1* and -2) [137]. 

Due in part to its relationship with cholesterol and cardiovascular disease, *ABCA1* is one of the most studied genes of ColoLipiGene signature. It is well known that curcumin enhanced cholesterol efflux by upregulating *ABCA1* expression through AMPK-SIRT1-LXRα signaling in THP-1 macrophage-derived foam cells [138]. It have been also described that hesperetin, a citrus flavonoid, increased *ABCA1* promoter and LXR enhancer activities in THP-1 macrophages [139]. Recently, dietary compounds from olive oil were tested for their capacity to enhance cellular ABCA1 protein level, and authors identified erythrodiol (Olean-12-ene-3b,28-diol) as an ABCA1 stabilizer [140]. Additionally, the mRNA and protein expression of LXRs and their target genes, including *ABCA1*, was significantly increased in macrophages stimulated with cineole [141]. The 1,8-Cineole (cineole), also known as eucalyptol or cajeputol, is a terpene oxide and a principal component of most eucalyptus oils, rosemary, and many other essential oils. Continuing in this line, other bioactive products like piperine (*Piper nigrum*) [142], silymarin (*Silybum marianum* L.) [143] and garlic-derived compounds [144], also modulate *ABCA1* expression. Nevertheless, studies in colorectal cancer models are needed in order to explore new therapeutic or preventive applications.

## 5. Concluding Remarks

Compelling evidence gained from epidemiological and experimental studies supports the crucial role of obesity, dietary patterns, gene-diet interactions and lipid metabolism in CRC prevention and prognosis. 

Despite all advances in early cancer diagnosis and in the development of new targeted therapies, still many tumors continue to be untreatable. This is mainly due to inter and intra-heterogeneity, both in primary lesions and distal metastasis. The integration of basic and translational nutritional research into the clinic is contributing to identifying groups of patients and subsequent strategies for personalized treatment and diet recommendations.

Precision nutrition opens a window of opportunity to integrate omics technologies with clinical advice. In particular, lipid metabolism is gaining interest in the scientific community as a bona-fide target in CRC. The ultimate goal should be to identify or generate bioactive compounds that directly or indirectly modulate lipid metabolic processes. The design of clinical trials that combine classical chemotherapeutic agents with bioactive products targeting lipid metabolism constitutes an unquestionable line of research in CRC treatment.

## Figures and Tables

**Table 1:** Associations between genetic variants in diet-nutrition-related genes and colorectal cancer (CRC) risk.

Gene Symbol	Gene Name	SNP		CRC Risk	Interactors	Reference
*MTHFR*	Methylenetetrahydrofolate reductase enzyme	rs1801133	Cys677Thr	Reduced	High folate intake	[100,101,102]
*GSTM1*	Glutathione S -transferase M1	-	Null Phenotype	Increased	-	[103,104]
*GSTT1*	Glutathione S -transferase T1	-	Null Phenotype	Increased	-	[103,104]
*APEX1*	Ascorbate peroxidase	rs1048945	Glu51His	Reduced	-	[111]
*PARP*	Poly ADP ribose polymerase	rs1136410	Val762Ala	Modifier of rs1048945	High-temperature cooked red meat	[111]
*FLT1*	Vascular endothelial growth factor receptor 1	rs678714		Reduced	Smoking	[112]
		rs2387632		Reduced	Animal protein intake	
*KDR*	Vascular endothelial growth factor receptor 2	rs6838752		Increased	Alcohol	[112]
*BMP4*	Bone morphogenetic protein 4	rs17563		Reduced	Smoking	[112]

**Table 2:** Associations between polymorphisms in lipid-metabolism-related genes and colorectal cancer.

Gene Symbol	Gene Name	SNP	CRC Cases	Controls	Model	CRC Risk	Measure of Risk	(95% CI)	*p*-Value	Reference
*HPGD*	Hydroxyprostaglandin dehydrogenase 15-(NAD)	rs2612656	1225	2032	Dom.	Increased risk of developing CRC	OR: 1.24	(1.07–1.44)	0.005	[130]
		rs8752	1225	2032	Dom.	Increased risk of developing CRC	OR: 1.22	(1.05–1.43)	0.009	[130]
*PLA2G6*	Phospholipase A2 group VI	rs4821737	1225	2032	Rec.	Increased risk of developing CRC	OR: 1.26	(1.06–1.50)	0.009	[130]
*TRPV3*	Transient receptor potential vanilloid 3	rs11078458	1225	2032	Rec.	Increased risk of developing CRC	OR: 1.32	(1.10–1.59)	0.003	[130]
*PTGER2*	Prostaglandin E receptor 2	rs17831718	1225	2032	Dom.	Reduced risk of developing CRC	OR: 0.73	(0.58–0.91)	0.006	[130]
*LIPC*	Hepatic triglyceride lipase	rs9652472	1780	1864	Log-add.	Increased risk of developing CRC	OR: 1.52	(1.20–1.92)	0.0005	[131]
*ACSL1*	Acyl-CoA synthetase 1	rs8086	284	-	Rec.	Increased risk of CRC relapse	HR: 3.08	(1.69–5.63)	0.046	[11]

Dom.: Dominant model of inheritance; Rec.: Recessive model of inheritance; Log-add: Log-Additive model; OR: Odds ratio; HR: Hazzard ratio; Ref: Reference; SNP: Single Nucleotide Polymorphism; CRC: Colorectal cancer; CI: Confidence interval.
